# Alterations in Cardiac Metabolism by *Trypanosoma cruzi* Infection: A Metabolomic Assessment by RPLC-MS and GC–MS

**DOI:** 10.1021/acsinfecdis.5c00819

**Published:** 2025-11-20

**Authors:** Hanna Carvalho de Sá, Breno Cardim Barreto, Maria Vitória Gomes das Neves, Maria Gabriela Sarah Santos, Carine Machado Azevedo Cardoso, Juliana Fraga Vasconcelos, Milena Botelho Pereira Soares, Gisele André Baptista Canuto

**Affiliations:** † Department of Analytical Chemistry, Institute of Chemistry, Federal University of Bahia, Salvador, BA 40170-115, Brazil; ‡ SENAI Institute of Innovation in Health Advanced Systems, SENAI CIMATEC, Salvador, BA 41650-010, Brazil; § Gonçalo Moniz Institute, 42509 FIOCRUZ, Salvador, BA 40296-710, Brazil

**Keywords:** *Trypanosoma cruzi*, Chagas disease, untargeted metabolomics, cardiomyopathy

## Abstract

Chagas disease (CD), caused by *Trypanosoma cruzi*, has been one of the leading causes of cardiac death in Latin America. Its pathogenesis and progression are still poorly understood. Thus, we performed an untargeted metabolomics analysis to understand the metabolic changes involved in the final acute phase of CD. Male mice’s chagasic hearts (60 days postinfection) were compared to healthy tissues. Two hundred and fifty-one significant metabolites or chemical classes were annotated. Disturbances in energy metabolism and dysregulation of amino acids were observed. Pathway analyses indicated increased inflammatory activity in infected individuals, as observed by eicosanoid (prostaglandin and thromboxane) changes. The accumulation of some sphingomyelins, correlated with myocarditis, suggests heart tissue damage from the infection. The metabolic changes observed contribute to understanding disease progression and the cardiac effects caused by the parasite, bringing new insights into the discovery and development of new therapies.

## Graphical abstract

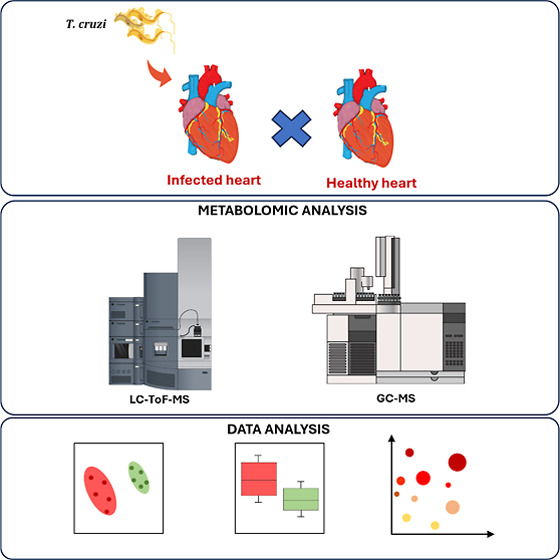

Chagas disease (CD), a parasitic infection caused by the protozoan parasite *Trypanosoma cruzi*, is classified by the World Health Organization (WHO) as a neglected tropical disease mainly endemic in Latin America.^1^ CD is classified according to two clinical stages, the acute phase (1–8 weeks postinfection) and the chronic phase. The first host–parasite interactions occur in the acute phase, and immunological events during this phase can impact disease progression. Thus, understanding the acute phase’s metabolic changes may help to determine how the infection will progress.^2^ In most cases, during the acute phase, the disease is asymptomatic or with mild symptoms such as fever, malaise, and, in the case of vector transmission, the presence of chagoma (a sign of entry of *T. cruzi* in the body); these symptoms, being nonspecific, make it difficult to detect the disease. Occasionally, the acute phase causes severe life-threatening myocarditis and meningoencephalitis, often in immunosuppressed patients or newborns. Failure to treat CD in the initial stage results in parasite persistence and progression to the chronic form of the disease, which is generally asymptomatic for years. In the long term, the disease affects the cardiac and digestive systems in about 40% of the patients.^3^

The pathogenesis of CD is not yet fully understood, and the existing etiological treatment is limited to Nifurtimox and Benznidazol. These drugs act only in the acute phase, aiming to alleviate symptoms or eliminate the parasite.^4^ Therefore, early diagnosis of the initial phase allows for the introduction of therapies that can effectively treat the infection. In this context, it is necessary to elucidate and understand the mechanisms involved in *T. cruzi* infection.

An approach that allows us to elucidate and understand the metabolic changes resulting from Chagas disease is metabolomics. Metabolomics consists of the study of changes in metabolites, intermediate or final products of metabolism in a biological sample, aiming to understand pathologies at the molecular level.^5^ It can be divided into untargeted metabolomics (comprehensive analysis of all altered metabolites) and targeted metabolomics (selective analysis of chemical classes or metabolites). The metabolome (set of all metabolites present in the organism) varies in physicochemical properties and concentrations. This poses a challenge in the analysis of metabolites, as it is not possible to cover the entire metabolome with a single analytical technique. The main analytical techniques applied in metabolomics studies are nuclear magnetic resonance (NMR) spectroscopy and mass spectrometry (MS). Due to the complexity of the samples processed, most studies use MS coupled to a separation technique, such as liquid chromatography, gas chromatography, or capillary electrophoresis, which allows the reduction of matrix effects.^6^ Additionally, more than one analytical platform can be used to increase the metabolic coverage.

Current studies of CD involving metabolomics have been investigating potential biomarkers in faeces^7^ and serum^8^ from animal models, as well as serum samples from humans.^9^ The effectiveness of existing treatments in serum or plasma from human^10,11^ and male mice^12^ is also found. Cardiac tissue presents itself as an interesting specimen for studying and understanding CD. In this sense, Hoffman et al. (2021) correlated metabolites already associated with CD with cardiac pathologies developed by mice in the chronic phase.^13^ McCall et al. (2017) and Dean et al. (2021) carried out studies involving spatial metabolomics to verify metabolic changes in different sections of the heart in animal models, in which the increase in glycerophosphocholines and reduction in acylcarnitines were correlated to a higher parasite concentration.^14,15^ Díaz et al. (2022) evaluated the metabolic differences in human cardiac tissue with heart failure secondary to Chronic Chagasic Cardiomyopathy (CCC).^16^ This interesting study showed alterations in critical antioxidant systems associated with the chronic inflammatory process of the disease. To date, only one study using cardiac tissue and plasma samples from female mice has evaluated CD in the acute phase at 14- and 21-days postinfection (DPI). The main changes were observed in heart tissues with glucose uptake, the TCA cycle, sorbitol, and fatty acid pathways affected by the infection.^17^ This study provided important insights into understanding the pathogenesis of the acute form; however, further investigations are necessary to obtain a more comprehensive assessment of the nonpolar fraction of the metabolome. Thus, in this work, the metabolic changes caused by Chagas Disease in the final of acute phase (60 DPI) were evaluated in cardiac tissues by multiplatform untargeted metabolomics, covering the polar and nonpolar metabolome.

## Results and Discussion

### Studied Groups and Parasite Infection

One of the main clinical manifestations of Chagas disease is the development of cardiovascular disorders such as severe myocarditis, heart failure, and CCC.^3^ However, there is still little understanding of the infectious mechanisms that result in the development of these pathologies. Thus, this study employed an untargeted metabolomics approach using a multiplatform approach to understand the metabolic alterations resulting from CD in cardiac tissue. The cardiac tissue samples from mice were divided into two groups, one of healthy animals (named CTR) and the other infected with *T. cruzi* (named IFC), in which the animals were euthanized 60 DPI, characterizing the final of the acute phase of CD (Figure S1 in the Supporting Information).

Sections of the left ventricle were stained with hematoxylin–eosin (H&E) and subjected to morphometric analysis of the inflammatory infiltrate. At 60 days post–*T. cruzi* infection, a multifocal inflammatory infiltrate with a predominance of mononuclear cells was observed. The number of inflammatory cells was significantly higher in chagasic animals compared to uninfected controls, indicating the persistence of a myocardial inflammatory response in the acute phase of infection (Figure 1). Immunostaining using anti-*T. cruzi* antibodies showed the presence of parasite nests in the cardiac tissue (Figure 2).

**Figure 1:**
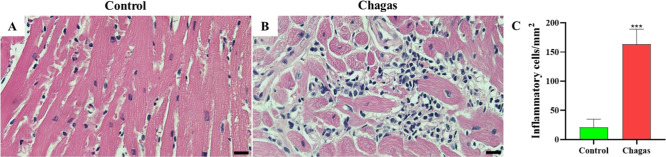
Morphological analysis in the hearts of uninfected and chagasic mice at 60 days post infection. (A,B) Representative micrographs of hematoxylin and eosin-stained heart sections of uninfected (control) and chagasic mice at 60 days following infection. (C) Inflammatory cells quantified by morphometric analysis. Values represent means ± SEM of 5 mice per group. ***P < 0.001 compared to control group; scale bars = 50 μm.

**Figure 2:**
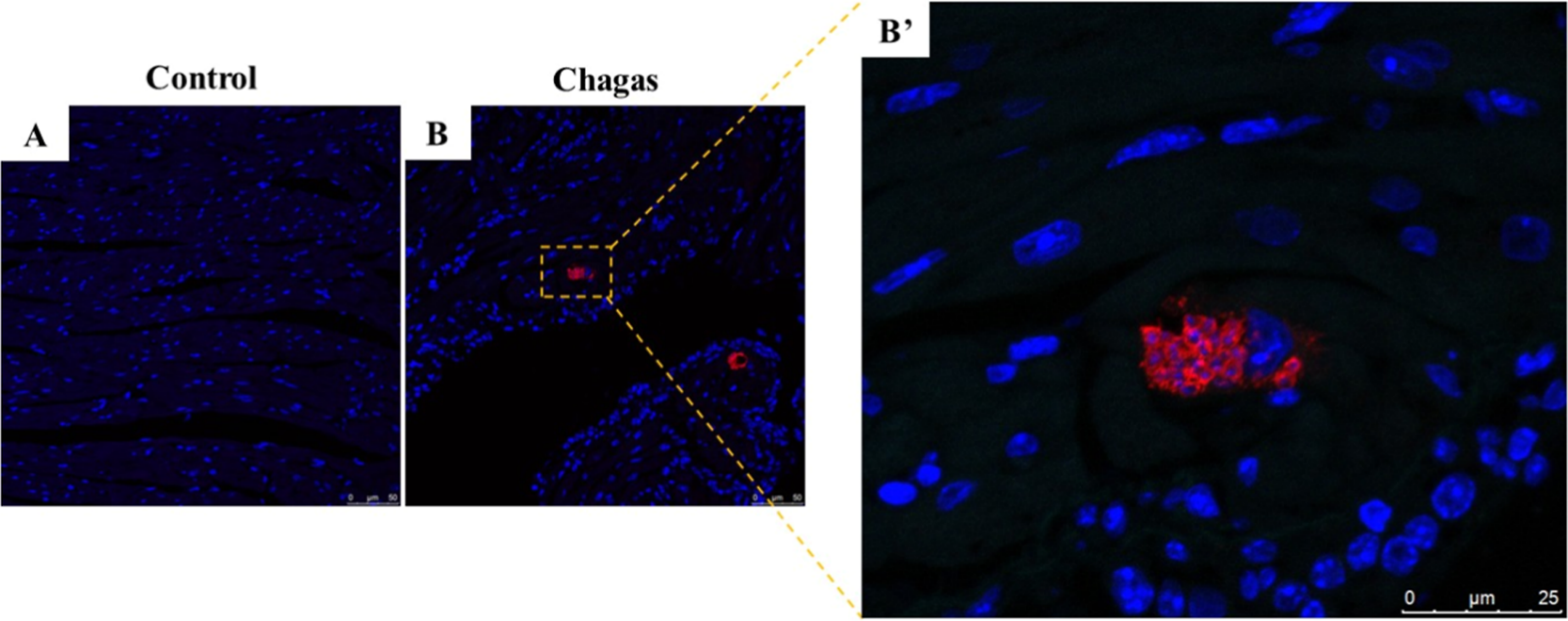
Immunofluorescence analysis in hearts of chagasic mice and uninfected controls. (A) Heart sections from uninfected control mice and (B and B′) chagasic mice. Samples were stained with DAPI (blue) and with anti-*T. cruzi* serum (red; 1:500); images were analyzed by confocal microscopy. Scale bars = 50 μm (A and B) and 25 μm (B′).

## Metabolomics Analysis

Metabolite extractions with organic solvents associated with mechanical apparatus for tissue rupture and greater metabolite efficiency were applied.^18^ Data were obtained using two analytical platforms: reversed-phase liquid chromatography–mass spectrometry (RPLC–MS) and gas chromatography–mass spectrometry (GC–MS), to cover the metabolome’s nonpolar/moderately polar and volatile portions, respectively. RPLC-MS analysis was performed in positive (ESI+) and negative (ESI−) ionization modes.

The data were processed in XCMS and MS-DIAL (processing parameters are presented in Table S1) and duly filtered and normalized in the MetaboAnalyst 6.0 platform. A filter step, removing data with relative standard deviation (RSD) > 30% in the quality control (QC) samples, was performed, in which 4912 (RPLC-ESI+), 2464 (RPLC-ESI−), and 91 (GC–MS) molecular features (MF) were obtained. The first inspection of the data involved grouping the quality control samples into multivariate models. QCs represent the average of the metabolome under study since they were prepared by mixing equal volumes of all studied samples. As can be seen in Figure S2, the unsupervised Principal Component Analysis (PCA) models demonstrate good grouping of the QCs, evidencing instrumental stability during data acquisition. Furthermore, the models also demonstrate a clear tendency of separation between the IFC and CTR groups, indicating differences in the extracted metabolome. Supervised PLS-DA (Partial Least Square-Discriminant Analysis) models for RPLC-MS data and OPLS-DA (Orthogonal Partial Least Square-Discriminant Analysis) for GC–MS data were built to find the discriminants. Figure 3 shows the supervised models comparing the IFC versus the CTR groups. The models presented good correlation (*R* ^2^ > 0.98) and prediction (*Q* ^2^ > 0.75) of the data. Permutation tests using the separation by distance (B/W) method, applying 1000 permutations, were performed, in which the models were validated with *p*-value = 0.037, 0.027, and 0.022 for RPLC-ESI+, RPLC-ESI–, and GC–MS, respectively. The differences between the studied groups and the search for discriminant molecular features were achieved by analyzing Variable Importance on Projection (VIP score >1.0). For RPLC-MS, 562 (ESI+) and 475 (ESI−) significant molecular features were found, and for GC–MS, 40 MFs.

**Figure 3:**
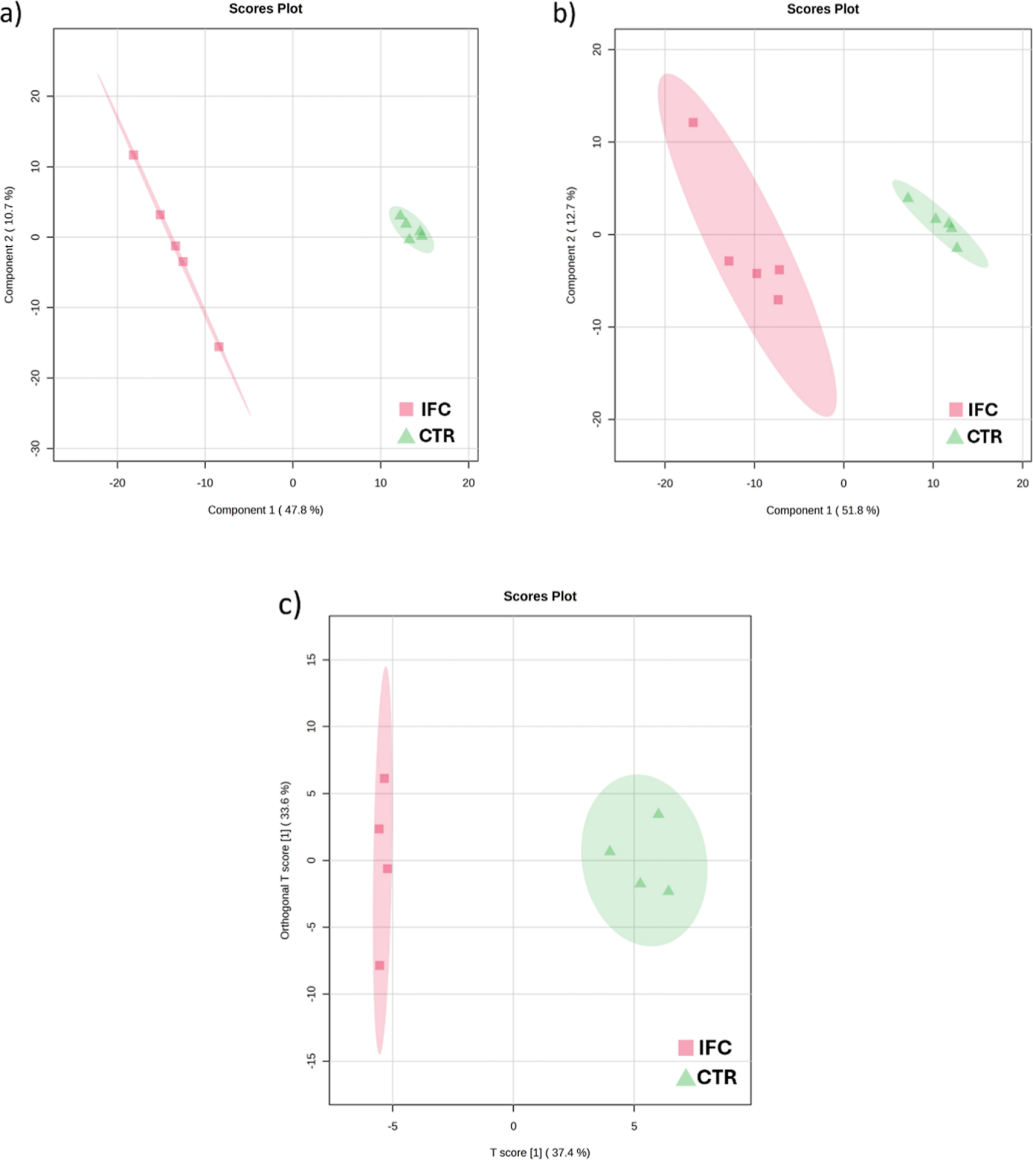
Supervised models built in MetaboAnalyst 6.0. (a) PLS-DA score plot of RPLC-MS (ESI+) normalized data (*R* ^2^ = 0.99; *Q* ^2^ = 0.94; and *p* = 0.037), (b) PLS-DA score plot of RPLC-MS (ESI−) normalized data (*R* ^2^ = 0.98; *Q* ^2^ = 0.87; and *p* = 0.027), and (c) OPLS-DA score plot of GC–MS normalized data (*R* ^2^ = 0.98; *Q* ^2^ = 0.75; and *p* = 0.026). Label: Infected (IFC)—red squares and Control (CTR)—green triangles.

Univariate evaluation of metabolomic data was performed using an in-house script. Normally distributed data were evaluated by the *t*-test and the F-test of homogeneous or heterogeneous variance. Data with non-normal distribution were statistically evaluated by the Mann–Whitney *U* test (nonparametric). Finally, to remove false positives, a False Discovery Rate (FDR) test was applied to data that presented a *p*-value < 0.05, in which MFs with FDR < 0.05 were considered significant. Thus, 524, 357, and 06 significant MFs were found for RPLC-ESI+, RPLC-ESI–, and GC–MS analyses, respectively.

The annotation (level 3 according to the Metabolomics Standard Initiative, MSI)^19^ of the RPLC-MS data was performed by searching the *m*/*z* in public databases (HMDB, LipidMaps, and KEGG), in which 228 metabolites or chemical classes were annotated, of which 146 for ESI+ and 82 for ESI–. The annotations were performed using the possible adduct generated in the corresponding ionization mode after carefully evaluating the chemical structure and the correlation with the type of sample used. Table S1 presents the metabolites annotated by RPLC-MS, in which fatty acids, glycerophospholipids, steroid lipids, and sphingolipids are highlighted. The MFs detected by GC–MS were annotated according to the spectral fragmentation patterns compared with libraries (FiehnLib and HMDB) in MS-DIAL software. Twenty-three metabolites were annotated (level 2 or 3, according to MSI). Table S1 presents the metabolites annotated by GC–MS, in which alterations are observed in the levels of carbohydrates, fatty acids, sterol lipids, organic acids, ester phosphates, and amino acids. Table S1 also presents fold change (FC) values. The FCs were calculated by the ratio between the mean intensities of the metabolites or classes in the IFC group and those in the CTR group. FC > 1.0 indicates an increase in the metabolite in the *T. cruzi*-infected group compared to the control. In contrast, FC < 1.0 indicates a decrease in the level of this metabolite or class in the infected group.

Figure 4 compiles all chemical classes altered in this study. A Receiver Operating Characteristic (ROC) analysis was performed on metabolites with significant alterations to determine the accuracy of separation between groups and characterization of the compound as a potential biomarker. The estimated probability of correct data classification was assessed by the area under the curve (AUC) value, in which AUC > 0.9 is considered highly predictive. Figure 5 presents information regarding the variations of relevant metabolites in the IFC (red) and CTR (green) groups and their respective AUC.

**Figure 4:**
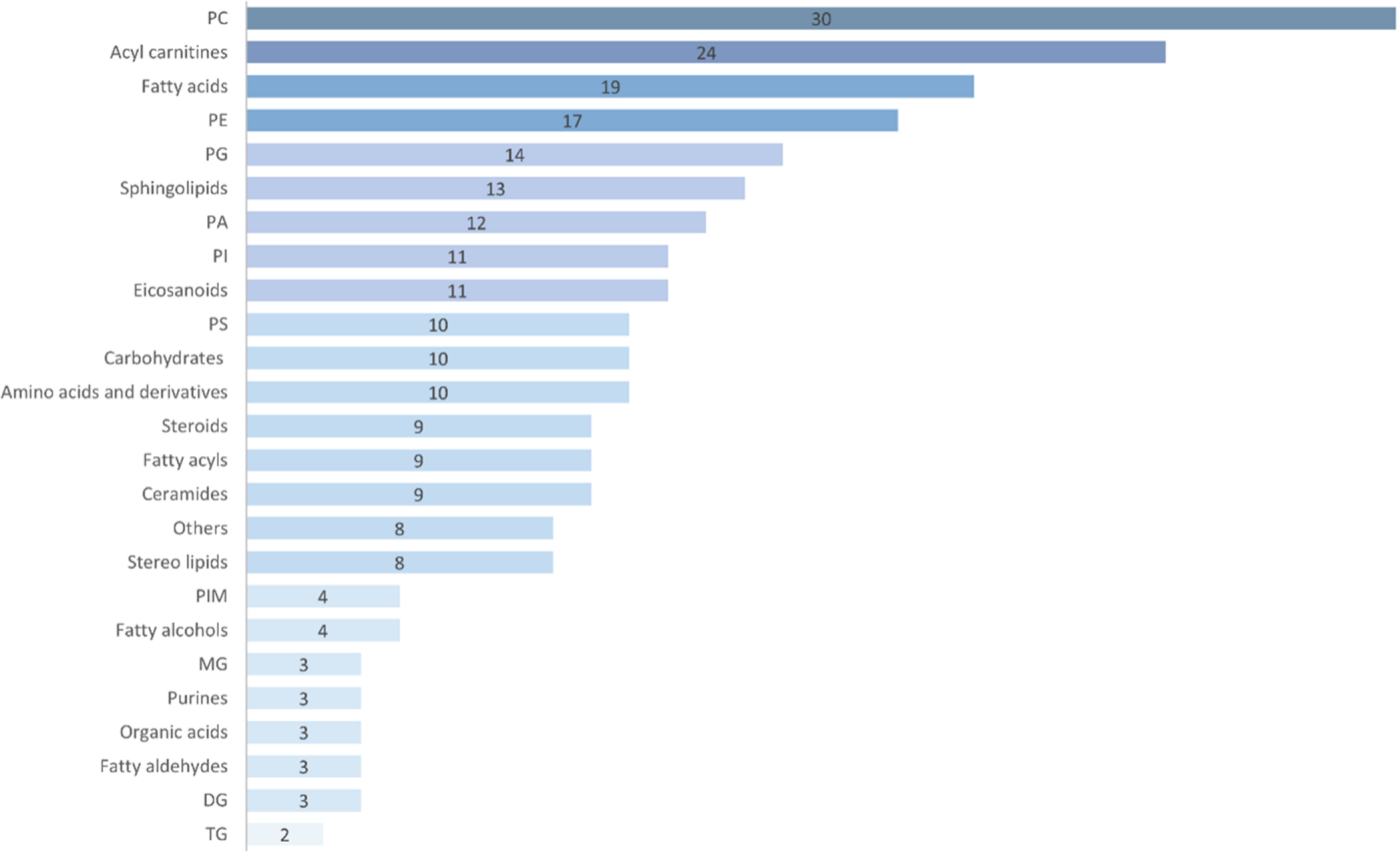
Bar chart of significantly altered metabolite classes. The number indicates the number of altered molecular features belonging to each class. PC—glycerophosphocholines, PE—glycerophosphoethanolamine, PG—glycerophosphoglycerols, PA—glycerophosphates, PI—glycerophosphoinositols, PS—glycerophosphoserines, PIM—glycerophosphoinositolglycans, MG—monoacylglycerols, DG—diacylglycerols, and TG—triacylglycerols.

**Figure 5:**
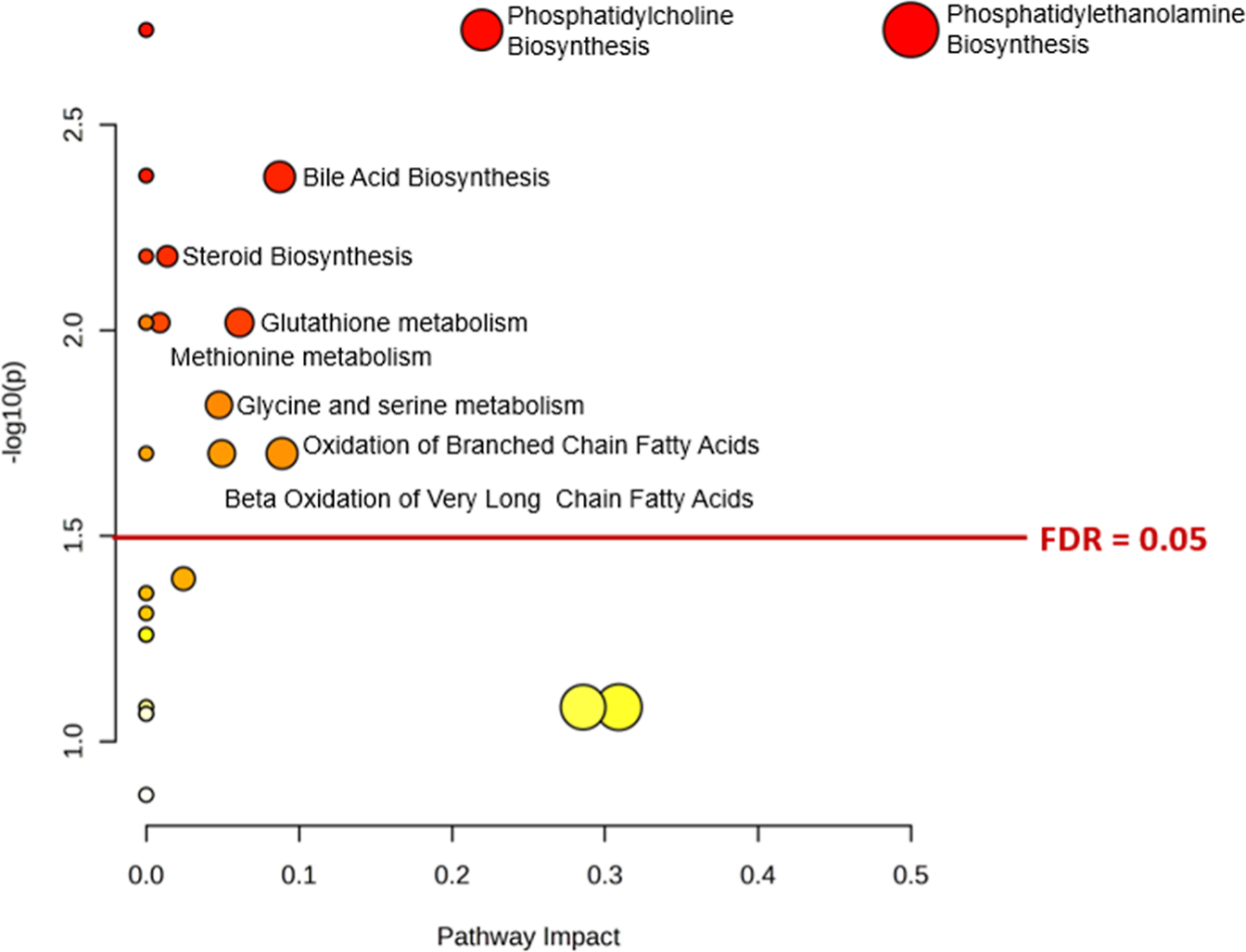
Pathway enrichment analysis of significantly annotated metabolites by GC–MS based on The Small Molecule Pathway Database (SPMDB). Red and orange dots represent significantly altered pathways based on *p*-value and FDR < 0.05. Yellow dots represent no significant pathways.

### Metabolic Pathway Analysis

Due to the large number of significantly altered metabolites belonging to different chemical classes, metabolic pathway analyses were performed to help understand the metabolic changes observed in the study.

The significant metabolites annotated by GC–MS were correlated with metabolic pathways through pathway analysis using the SMPDB (The Small Molecule Pathway Database) evaluated in mouse metabolism (*Mus musculus*). Figure 5 shows the enrichment analysis result, in which nine metabolic pathways with high impact were altered significantly (FDR < 0.05). Phosphatidylethanolamine Biosynthesis (impact = 0.50) and Phosphatidylcholine Biosynthesis (impact = 0.22) were the pathways with the most significant alterations related to changes in phosphoethanolamine levels. Phosphoethanolamine was found to be increased with *T. cruzi* infection (FC = 2.85 and AUC = 0.88, Figure 6a).

**Figure 6:**
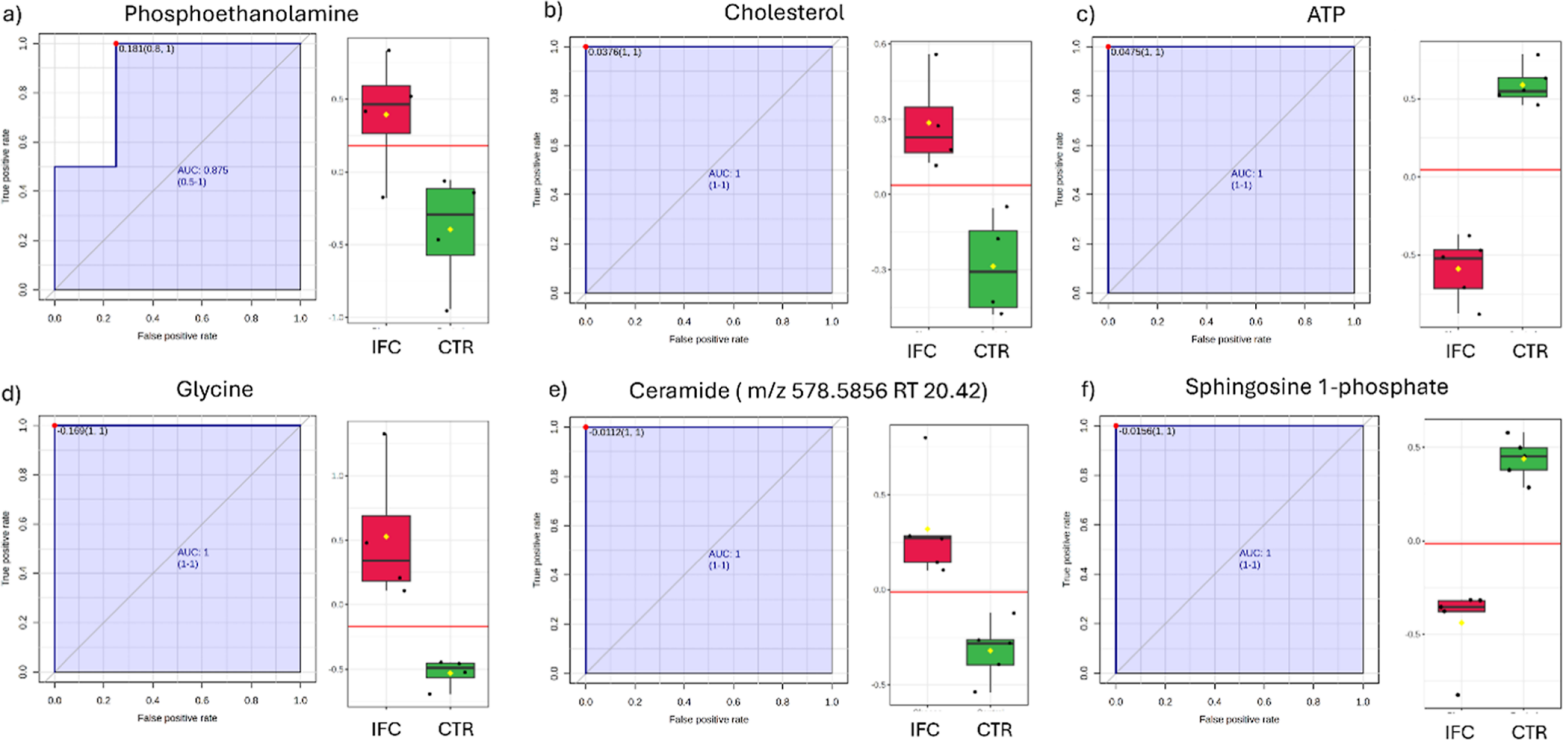
Univariate ROC curves and box plots: (a) phosphoethanolamine (FC = 2.85; phosphatidylethanolamine biosynthesis and phosphatidylcholine biosynthesis), (b) cholesterol (FC = 1.60; bile acid biosynthesis and steroid biosynthesis), (c) ATP (FC = 0.29), (d) glycine (FC = 7.05; sphingolipid metabolism), (e) ceramide (*m*/*z* 578.5856 RT 20.42) (FC = 7.49; sphingolipid metabolism), and (f) sphingosine 1-phosphate (FC = 0.58; sphingolipid metabolism).

The same alteration was previously reported by Girónes et al. (2014) and was correlated with tissue remodeling during infection.^17^ Alterations in the phosphatidylethanolamine and phosphatidylcholine biosynthesis pathways have been associated with key components of lipid metabolism and cell membrane structure.^20^ Other pathways with notable alterations are bile acid biosynthesis and steroid biosynthesis (Figure 5), both correlated mainly with changes in cholesterol levels (FC = 1.60 and AUC = 1.0, Figure 6b). In addition, metabolic pathways involved in central metabolism showed dysregulation, such as the amino acid pathways (glutathione metabolism, methionine metabolism, and glycine and serine metabolism) and fatty acid degradation (oxidation of branched chain fatty acids and beta oxidation of very long chain fatty acids).

Due to the limitations of structural confirmation, especially in the differentiation of isomers and adducts with the same molecular mass, the RPLC-MS data were evaluated by functional analysis using the GSEA (Gene Set Enrichment Analysis) algorithm.^21^ GSEA allows pathway analysis without the identification of metabolites, being an advantageous method for application in global metabolomics data, in which the information on *m*/*z*, RT, and peak area of significant molecular features was included in the processing. The analysis was performed separately for each ionization mode, using the KEGG and BioCyc libraries in MetaboAnalyst 6.0. The pathway enrichment analysis allowed for the finding of seven significantly altered metabolic pathways (Table 1).

**Table 1:** Significant Altered Pathways for Functional Analysis of RPLC-MS Data

pathway name	hits	sig hits	GSEA^a^ p	ionization mode
arachidonic acid metabolism	40/43	8	0.019	negative
porphyrin metabolism	6/31	3	0.020	negative
purine metabolism	6/71	3	0.014	negative
steroid hormone biosynthesis	19/87	4	0.016	negative
one carbon pool by folate	1/26	1	0.004	negative
carnitine shuttle	23/72	17	0.024	positive
tyrosine metabolism	3/42	2	0.023	positive

a GSEA, Gene Set Enrichment Analysis.

### Energy Metabolism

Pathway enrichment analysis indicated significant alterations in fatty acid (FA) metabolism, in which an increase in the level of these metabolites was observed with *T. cruzi* infection. FA metabolism is involved in energy metabolism, a process of adenosine triphosphate (ATP) harvesting energy.^22^ In this study, chagasic hearts showed decreased ATP levels (FC = 0.29 and AUC = 1.0, Figure 6c and Table S1) compared to the control group. Previous proteomic studies have reported alterations in energy metabolism pathways due to decreased expression of enzymes involved in FA metabolism.^23^ Reduced FA metabolism could contribute to a decrease in the adenosine triphosphate levels. However, this is not the only source of cellular energy generation. In addition to FAs, lactate, glucose, ketones, and amino acids are responsible for ATP formation via oxidative phosphorylation or glycolysis.^24^ ATP is one of the main energy sources for living beings, and maintaining cardiac functions requires large amounts of this metabolite. ATP dysregulation affects the energy supply to cardiomyocyte cells, which has been associated with heart failure.^24,25^ Also, the increased levels of FAs and accumulation of TCA cycle substrates malate and lactate, the latter a precursor of pyruvate, found in this study, may be associated with mitochondrial dysfunctions. Such dysfunctions may affect the metabolic flexibility of the heart, especially in conditions that lead to its failure, due to low ATP production.^24^

### Arachidonic Acid Metabolism

Functional analysis of RPLC-MS data indicated significant alterations in arachidonic acid (AA) metabolism. The increase in species belonging to AA metabolism has been associated with increased synthesis of prostaglandins due to the parasite incorporating host AA and releasing prostaglandins.^26^ Annotated metabolites associated with prostaglandins and thromboxane (Tx) increased in chagasic hearts in this investigation. Some studies involving Chagas disease have suggested that alterations in the levels of eicosanoids, such as prostaglandins and Tx, are related to the presence of protozoa in cardiac tissue.^14^ These compounds are released by *T. cruzi* regulate host responses to parasitic invasion, increasing inflammatory activity and acting to control disease progression. Therefore, prostaglandins and Tx have been associated with host survival in the acute phase and the transition of infection to the chronic phase.^27^ Different eicosanoids present increased levels during the acute phase, whereas in the chronic phase, only thromboxane A2 is expected to increase. Thus, these changes could be potential markers of the phase transition of the disease.^27^ The abundance of eicosanoids has also been associated with different pathologies, such as myocardial inflammation, thrombosis, and cellular dysfunctions.^27^ Furthermore, these metabolites are identified as responsible for the increased inflammatory activity in CD. In this study, the annotated eicosanoids present high levels in the chagasic group (Table S1, Supporting Information), with fold changes ranging from 1.78 to 22.25.

### Sphingolipids

In addition to dysregulation in the AA pathway, the inflammatory activity in CD is also impacted by sphingolipid metabolism. Our results indicate an increase in some sphingolipids, especially sphingomyelins (Table S1). Additionally, dysregulation in amino acid metabolism was influenced by the increase in the level of glycine (FC = 7.05 and AUC = 1.0, Figure 6d) in the chagasic group. Amino acids contribute to a higher concentration of sphingolipids, which are generated by the association of an amino acid with an acyl-CoA group.^28^ The increase and accumulation of sphingolipids, such as ceramides and sphingomyelins, have been correlated with cases of myocarditis;^28^ and they have been suggested to be responsible for tissue damage in cardiometabolic diseases.^29^ Our results showed several deregulations on the metabolism of free sphingoid bases (Figure 7), all metabolites showed an increase in the IFC group (such as ceramide *m*/*z* 578.5856, FC = 3.40, Figure 6e) except sphingosine 1-phosphate (S1P) (FC = 0.58, Figure 6f). Despite their close structural similarity, ceramide, sphingosine, and S1P play distinct and often opposing roles in cellular physiology. While ceramide and sphingosine are generally pro-apoptotic and inhibit cell proliferation, S1P promotes cell survival and growth, highlighting the importance of the ceramide/S1P rheostat in determining cell fate.^30^ In the context of *T. cruzi* infection, ceramide plays a pivotal role in parasite pathogenicity. Studies have shown that ceramide-rich glycolipids from *T. cruzi* can synergize with host IFN-γ to induce macrophage apoptosis, potentially facilitating parasite dissemination.^31^ This imbalance may contribute to cardiac damage and disease progression, underscoring the potential of targeting sphingolipid pathways in therapeutic strategies for Chagas cardiomyopathy.

**Figure 7:**
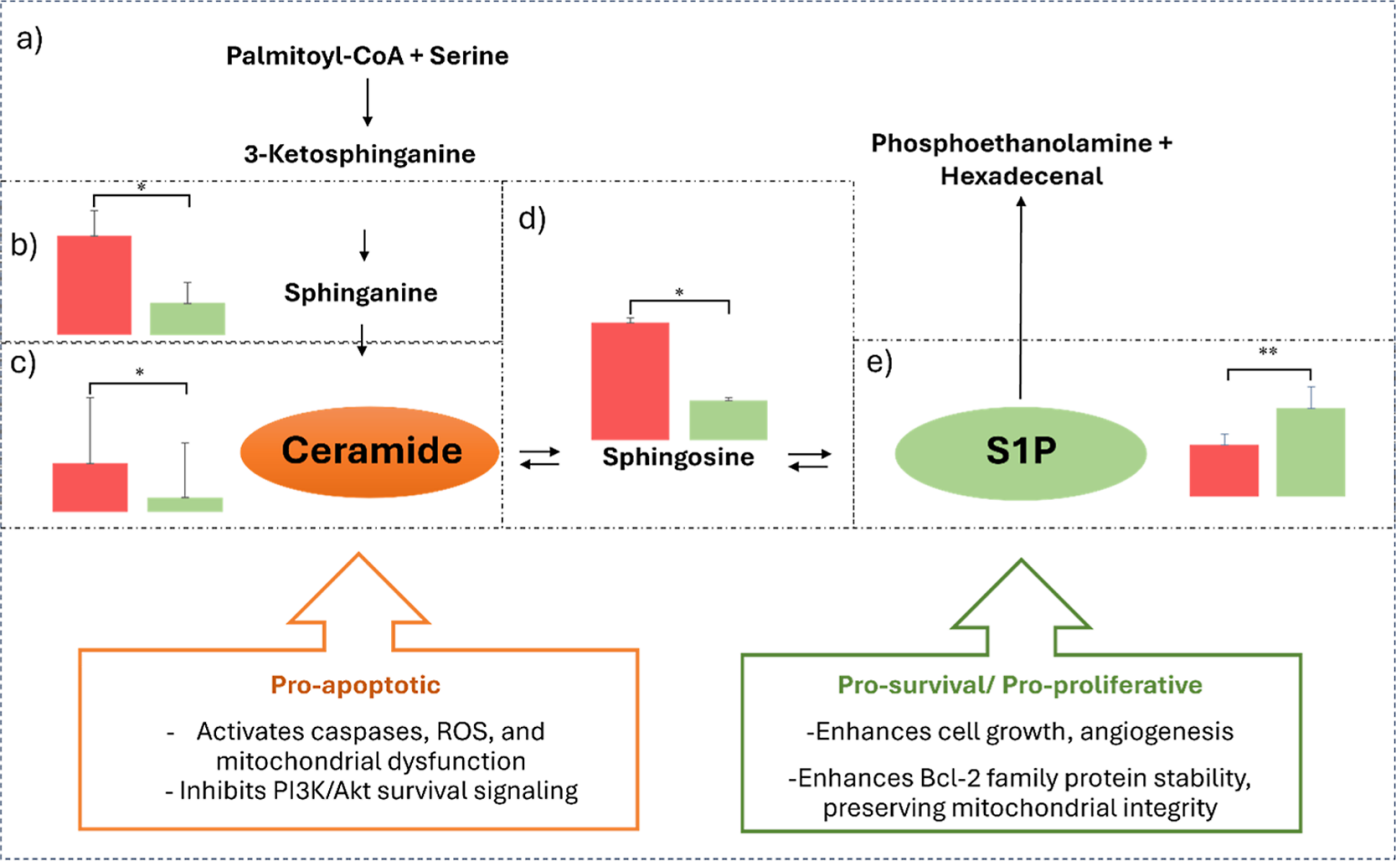
Semiquantitative analysis of sphingolipid metabolites was performed in the acute chagasic heart of C57Bl/6 mice (red bars), compared to noninfected (healthy) mice (green bars). A scheme of the sphingolipid pathway illustrating some steps: condensation of serine and palmityl-CoA (a), followed by a conversion to sphinganine (b) and ceramide production (c). Ceramide can be hydrolyzed by ceramidase to sphingosine (d), which is phosphorylated by sphingosine kinases to produce sphingosine 1-phosphate (S1P) (e). S1P is irreversibly degraded by sphingosine-1-phosphate lyase, which cleaves S1P into two products: phosphoethanolamine and a long-chain fatty aldehyde (such as hexadecenal). The ceramide/S1P rheostat is important to determine cell fate. Label: *p value < 0.05; **p value < 0.05, and VIP > 1.0.

## Conclusions

Multiplatform metabolomics (such as RPLC-MS and GC–MS) is a powerful tool for characterizing a larger portion of the metabolome and was applied here. Our results demonstrated interesting metabolic changes produced in the cardiac tissue of mice due to infection by *T. cruzi*. Changes in energy metabolism, arachidonic acid, and sphingolipids may be associated with cardiac inflammatory processes, resulting in cardiomyopathies and heart failure. These findings confirm speculations already observed in the literature and provide new insights for a better understanding of the consequences caused to cardiac tissue and consequent failure due to infection. Additional studies in complementary analytical platforms such as NMR and hydrophilic liquid interaction chromatography (HILIC), together with larger sampling, may complement the metabolic information acquired here, contributing to a better characterization of the disease and the development of new treatment strategies.

## Materials and Methods

### Chemicals

Mass spectrometry grade high-purity solvents methanol and heptane were purchased from JT Baker (Mexico). Pyridine silylation grade was procured from Merck Millipore (U.S.A.). N,O-bis­(trimethylsilyl)­trifluoroacetamide (BSTFA) with 1% (v/v) trimethylchlorosilane (TMCS), *O*-methoxyamine hydrochloride, and tridecanoic acid methyl ester were purchased from Sigma-Aldrich (Germany). Dichloromethane (Qhemis, Brazil) and deionized water (Milli-Q system, U.S.A.) were also used.

### Animal Model and Sample Collection

This study was approved by the Instituto Gonçalo Moniz—Fundação Oswaldo Cruz (IGM-FIOCRUZ) Research Ethics Committee (protocol no. 006/2023).

Male C57BL/6 mice (*n* = 10), 8 weeks old, were used. The animals were kept under controlled temperature (20 ± 2 °C) and humidity (50%), with food and water ad libitum, and exposed to a constant 12 h light–dark cycle.

Mice were infected by intraperitoneal injection with 1000 trypomastigotes of the Colombian strain of *T. cruzi*, obtained by in vitro infection of the LCC-MK2 cell line. Parasitemia was assessed at different postinfection periods by counting the number of trypomastigotes in peripheral blood samples. Sixty days after infection (60 DPI), the animals were euthanized, and the hearts were collected by thoracotomy, frozen in liquid nitrogen, and stored at −80 °C until metabolite extraction. Within the sample (*n* = 10), five animals belonged to the Infected group and five to the Control group.

### Morphometric Analysis

After euthanasia, the hearts were removed, divided longitudinally into two parts, and immediately fixed in 10% formalin for histopathological analysis. The samples were dehydrated, embedded in paraffin, sectioned into 5 μm slices, and stained with hematoxylin–eosin (HE) for the quantification of inflammatory cells by optical microscopy at 400× magnification. Images were digitized using a color digital video camera (CoolSnap, Montreal, Canada) attached to a BX41 microscope (Olympus, Tokyo, Japan). Morphometric analyses were performed using Image Pro Plus v.8.0 software (Media Cybernetics, San Diego, CA). Five representative images were acquired from regions of more intense inflammation for each mouse, and the inflammatory cells were manually counted in each photomicrograph. Counts from different fields were averaged, and the mean value was obtained for each animal. Data are expressed as the mean ± standard deviation for each experimental group.

### Immunofluorescence Analysis

Cardiac tissue fragments obtained from mice were fixed in 10% formalin and embedded in paraffin for slide preparation. Paraffin sections (3 μm) were deparaffinized in xylene and rehydrated in a graded alcohol bath. Antigen retrieval of the sections was performed in a bath heated to 98 °C in citrate buffer (pH 6.0). After cooling, the slides were washed with PBS, permeabilized with Triton (0.1%), and blocked for nonspecific sites with Cas-block (Thermo Fisher Scientific). The anti-*T. cruzi* serum (1:500) produced in hamsters was diluted in PBS/1% BSA and incubated overnight at 4 °C. The next day, after washing, the secondary antibody antihamster IgG AlexaFluor 568 conjugated (1:800; Life Technologies) was added and followed by a 1-h incubation period at room temperature. Slides were mounted by using a Fluoroshield mounting medium with DAPI (Sigma-Aldrich). Images were obtained using a TCS SP8 spectroscopic confocal microscope (Leica).

### Metabolite Extraction

The samples were thawed in an ice bath, and the biomass of the hearts was determined by weighing. The extraction of metabolites was adapted from a methodology previously published by Want et al. (2013).^32^ Briefly, the samples were homogenized in a sample homogenizer system (TissueLyser II, QIAGEN), to which 1.5 mL of 50% cold methanol and one tungsten carbide bead (3 mm, QIAGEN) were added. The extraction was performed in five cycles: 15 Hz for 1:30 min; 20 Hz for 1:30 min; 25 Hz for 2 min; 30 Hz for 2 min; and 30 Hz for 2 min, followed by centrifugation at 10,000*g* for 10 min at 4 °C (MIKRO 200R, Hettich Zentrifugen). After collecting the supernatant, the sample was re-extracted by adding 1.6 mL of cold dichloromethane/methanol (3:1). The second extraction in the homogenizer system consisted of three cycles of 30 Hz for 2 min each. The extracts were centrifuged again (10,000*g* for 10 min and 4 °C), and the supernatants were combined with those from the first extraction step. Two aliquots (200 μL) of each extract were added to vials. Quality control (QC) samples were prepared by mixing the same amounts of all samples under investigation. A blank sample was prepared using the solvents and processes applied to the tissue sample extraction in the absence of the latter. All extracts, QCs, and blanks were completely dried using a lyophilizer Lyoquest at −85 °C (Telstar) for subsequent treatment and appropriate dissolution to be employed in different analytical techniques. Samples were analyzed randomly, and QC samples were evaluated throughout the analytical sequence to attest to instrumental stability.

### GC–MS Derivatization

The derivatization of the samples used a methodology adapted from Canuto et al. (2017).^33^ The oximation step involved the addition of 20 μL of *O*-methoxyamine in pyridine (15 mg mL^–1^) to the dried extract. After homogenization for 10 s in an ultrasonic bath (Cristófoli, Brazil) and vortexing for 10 s (KASVI, Brazil), the reaction occurred at room temperature in the dark for 90 min. The silylation step was performed by adding 20 μL of BSTFA and 1% TMCS, followed by homogenization (sonication and vortexing for 10 s each). The reaction proceeded at 40 °C for 30 min. After cooling the samples, 120 μL of tridecanoic acid methyl ester (10 mg mL^–1^ in heptane), an internal standard, was added to the derivatized extract and analyzed by gas chromatography–mass spectrometry (GC–MS). All samples, QCs, and blanks were individually derivatized.

### RPLC–MS Analysis

The dried extracts (samples, QCs, and blanks) were resuspended in 120 μL of 50% methanol. The reversed phase liquid chromatography–mass spectrometry (RPLC-MS) analysis was performed using a liquid chromatography system (ACQUITY UPLC H-Class, Waters) coupled to a time-of-flight (ToF) mass spectrometer via electrospray ionization (ESI) (XEVO-G2-XS ToF, Waters). Separation was performed on a C18 column (2.1 mm × 50 mm, 1.7 μm, Acquity UPLC BEH, Waters), maintained at 40 °C. The mobile phase was composed of (A) deionized water with 0.1% formic acid (F.A.) and (B) methanol with 0.1% F.A. The elution gradient was: 25–75% B in 10 min, 75–90% B in 5 min, 90–95% B in 7.5 min, 95–25% B in 1.5 min, and 25% B in 6 min for column conditioning. The flow rate was 0.5 mL/min. The MS was operated in positive (ESI+) and negative (ESI−) ionization modes, in which 600 °C solvation temperature and 3.0 kV were applied at the ESI+ interface, and 500 °C solvation temperature and 2.50 kV were applied for the ESI–. The mass spectra were acquired in centroid mode in a full scan (100–1200 Da). Masslynx software (version 4.1) was used for instrument adjustment, operation, and data acquisition.

### GC–MS Analysis

GC–MS analysis was performed using a gas chromatography system (7820 A, Agilent Technologies, Santa Clara, CA, USA) coupled to a single quadrupole mass spectrometer (5977E MSD, Agilent Technologies, CA, USA) using a previously published method.^34^ Briefly, the separation was performed using a DB5-MS column (30 m length, 0.32 mm inner diameter, 0.25 μm film composed of 95% dimethylpolysiloxane/5% diphenyl polysiloxane, Agilent Technologies). Helium was used as a carrier gas at 1.0 mL min^–1^ flow rate. The injection temperature was kept at 250 °C, and the samples were injected with a 1:10 split using He at 10 mL min^–1^. The initial oven temperature was maintained at 50 °C, with an increase (10 °C/min) to 325 °C. The MS was operated at 290 °C for the transfer line, 230 °C for the filament source, and 150 °C for the quadrupole. The electron ionization source used −70 eV energy. The MS was operated in full scan mode (50–600 *m*/*z*). MassHunter Qualitative Analysis B.07.01 software (Agilent Technologies) was used for instrument operation and data acquisition.

### Data Processing and Statistical Analysis

RPLC-MS raw data were converted into *.mzML, using the ProteoWizard software (version 3.0.22155), while GC–MS raw data were converted into *.ABF files in Reyfics ABF converter 4.0 (https://www.reifycs.com/abfconverter/). For RPLC-MS data extraction, three QC samples were processed using Isotopologue Parameter Optimization (IPO, version 1.22.1), running at RStudio (version 4.2.0) in order to find the best data processing parameters. The optimized parameters (presented in Table S2 in Supporting Information) were applied for data matrix extraction in the XCMS software (version 3.18.0), running on the RStudio platform (version 4.2.0). For GC–MS data processing, peak detection, spectrum deconvolution, and alignment were performed on MS-DIAL (ver. 5.3.240719), and the parameters were evaluated manually using QC samples. The best parameters are presented in the Supporting Information (Table S3). The extracted data matrices were evaluated by multivariate and univariate statistical methods to find the molecular features responsible for differentiation between the infected and control groups.

Multivariate analyses were performed in MetaboAnalyst 6.0 (https://metaboanalyst.ca/), in which PCA (Principal Component Analysis) and PLS-DA (Partial Least Squares Discriminant Analysis) models were built. Discriminant molecular features were selected by VIP (Variable Importance in Projection) score (VIP > 1.0). GC–MS data was normalized by biomass, intensity of internal standard, and median. RPLC-MS data was normalized by interquartile and median for ESI+ and ESI–, respectively. Log transformation and Pareto scaling were applied to the data. Univariate analyses were performed using an in-house script in RStudio (version 4.2.0). Initially, a normality test (Shapiro–Wilk) was applied, followed by a nonparametric (Mann–Whitney *U* test) or parametric tests (homogeneous or heterogeneous variance). Finally, a False Discovery Rate (FDR) was applied to the data, in which molecular features with FDR < 0.05 were considered significant.

### Metabolite Annotation and Pathway Analysis

Metabolite annotation of RPLC-MS data, the significant *m*/*z*’s were searched in the integrated platform CEU Mass Mediator (http://ceumass.eps.uspceu.es/), in which HMBD, PubChem, LipidMaps, and KEGG were chosen as public databases. Adducts [M+H]^+^, [M+Na]^+^, [2M+H]^+^, [M–H]^−^, and [M+HCOOH–H]^−^ were selected for ESI+ and ESI– data, respectively, with 5 ppm of maximum mass error. For GC–MS data was achieved based on the spectral fragmentation pattern and retention time, using the FiehnRI and MoNA HMDB databases on MS-DIAL software.

Pathway analyses were carried out with significant annotated metabolites or molecular features in MetaboAnalyst 6.0. For RPLC-MS data, functional analysis was achieved by GSEA (Gene Set Enrichment Analysis), based on molecular features information (*m*/*z*, RT, and peak area), and the pathways were searched on KEGG (Kyoto Encyclopedia of Genes and Genomes) and BioCyc databases for *M. musculus*. For GC–MS data, the *M. musculus* SMPDB database was used. Pathways that presented a *p* value <0.05, verified by FDR, were considered significant.

## Supplementary Material





## Notes

Parasitemia, data processing parameters, and multivariate models (PDF)
Altered metabolites or chemical classes (XLSX)

## Abbreviations

AA: arachidonic acid
AUC: area under the curve
CCC: Chronic Chagasic Cardiomyopathy
CD: Chagas disease
CTR: control group
DG: Diacylglycerols
DPI: days postinfection
ESI: electrospray
FA: fatty acid
FC: Fold Change
FDR: False Discovery Rate
GC-MS: Gas Chromatography–Mass Spectrometry
GSEA: Gene Set Enrichment Analysis
HILIC: Hydrophilic Liquid Interaction Chromatography
IFC: infected group
MF: molecular features
MG: Monoacylglycerols
MS: Mass Spectrometry
MSI: Metabolomics Standard Initiative
NMR: Nuclear Magnetic Resonance
OPLS-DA: Orthogonal Partial Least Square-Discriminant Analysis
PA: Glycerophosphates
PC: Glycerophosphocholines
PCA: Principal Component Analysis
PE: Glycerophosphoethanolamine
PG: Glycerophosphoglycerols
PI: Glycerophosphoinositols
PIM: Glycerophosphoinositolglycans
PLS-DA: Partial Least Square-Discriminant Analysis
PS: Glycerophosphoserines
QC: quality control
ROC: Receiver Operating Characteristic
RPLC-MS: Reversed-Phase Liquid Chromatography–Mass Spectrometry
RSD: relative standard deviation
S1P: sphingosine 1-phosphate
SPMDB: The Small Molecule Pathway Database
TG: Triacylglycerols
Tx: thromboxane
VIP: Variable Importance on Projection
WHO: World Health Organization.
