# Supplementary material for: Alterations in Cardiac Metabolism by Trypanosoma cruzi Infection: A Metabolomic Assessment by RPLC-MS and GC–MS
Source: ACS Infect Dis. 2025 Nov 20;11(12):3620–30. doi: 10.1021/acsinfecdis.5c00819 (PMC12706771; doi:10.1021/acsinfecdis.5c00819)
Supplement: Supplementary file 1 [file id5c00819_si_001.pdf]

Supporting Information

Alterations in cardiac metabolism by *Trypanosoma cruzi* infection: a metabolomic assessment by RPLC-MS and GC-MS

Hanna Carvalho de Sá[1], Breno Cardim Barreto[2,3], Maria Vitória Gomes das Neves[3], Maria Gabriela Sarah Santos[3], Carine Machado Azevedo Cardoso[3], Juliana Fraga Vasconcelos[3], Milena Botelho Pereira Soares[2,3], Gisele André Baptista Canuto[1]*

[1]Department of Analytical Chemistry, Institute of Chemistry, Federal University of Bahia, Salvador, BA, Brazil.

[2]SENAI Institute of Innovation in Health Advanced Systems, SENAI CIMATEC, Salvador, BA, Brazil.

[3]Gonçalo Moniz Institute, FIOCRUZ, Salvador, BA, Brazil.

*Corresponding author

Email adress: gisele.canuto@ufba.br (Canuto, G.A.B)

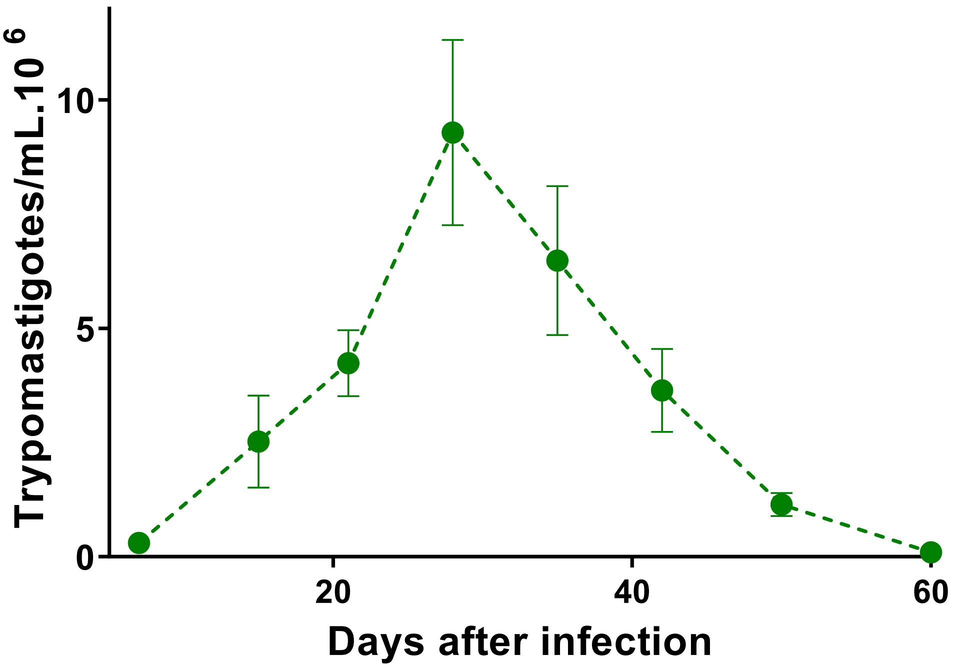

Figure S1. Parasitemia evolution during *T. cruzi* infection.

a)

b)

c)

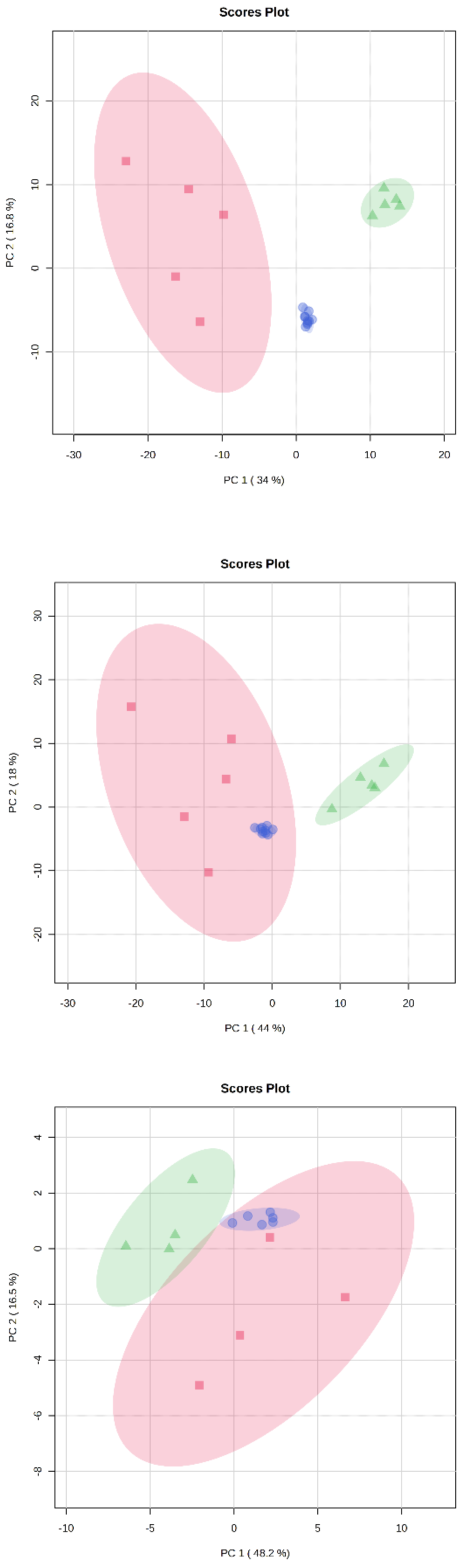

Figure S2. PCA models built in MetaboAnalyst 6.0. **a)** Scores plot of RPLC-MS (ESI+) biomass and quantile normalized data (PC1 = 34%; PC2 = 16.8%; ANOVA p = 0.001), **b)** Scores plot of RPLC-MS (ESI-) median normalized data (PC1 = 44%; PC2 = 18%; ANOVA p=0.001), and **c)** Scores plot of GC-MS Biomass and Internal Standard normalized data by (PC1 = 40.2%; PC2 = 16.5%; ANOVA p = 0.001). Label: Chagas – red squares; Control – green triangles; Quality Controls (QC) – blue dots.

Table S1. Optimized parameters evaluated by IPO for RPLC-MS (ESI+ and ESI-) data processing in XCMS.

XCMS Parameters								
Peak Detection			Alignment			Grouping		
	ESI+	ESI-		ESI+	ESI-		ESI+	ESI-
method	centWave		method	obiwarp		method	density	
peakwidth (min, max)	(11.844, 74.6)	(26.8, 48.4)	plottype	none		bw	12,4	0.879
ppm	20		distFunc	cor_opt		mzwid	0.0138	0.009
noise	0		profStep	1		minfrac	0.82	0.905
snthresh	10		center	2		minsamp	1	
mzdiff	-0,0296	-0,0175	response	1		max	50	
Prefilter (min, max)	(3, 100)		gapInit	0,64	0,286			
mzCenterFun	wMean		gapExtend	2.7				
integrate	1		factorDiag	2				
fitgauss	FALSE		factorGap	1				
verbose.columns	FALSE		localAlignment	0				

Table S2. MS-DIAL parameters for GC-MS data processing.

MS-DIAL parameters		
**Measurement parameters**	Ionization type	Hard ionization (GC-MS)
	Separation type	Chromatography
	Data type	Centroid data
**Data collection**	Mass range	40-600
	Retention time range	0-30
	Number of threads	3
**Peak detection**	Minimum peak height	50000
	Mass slice width	0.5
	Mass accuracy for centroiding	0.5
	Smoothing method	Linear weight moving average
	Smoothing level	2
	Average peak width	10
**Spectrum deconvolution**	Sigma window value	0.5
	EI spectra cut off	10
**Identification/ Annotation**	Retention time setting	RT
	RT tolerance	1
	m/z range begin	40
	m/z range end	600
	m/z tolerance	0.5
	EI similarity cut off	70
	Dot product score cut off	60
	Identification score cut off	70
**Alignment**	Reference file	QC5
	RI or RT	RT
	Retention time tolerance	0.5
	EI similarity tolerance	70%
